# Spot delivery error predictions for intensity modulated proton therapy using robustness analysis with machine learning

**DOI:** 10.1002/acm2.13911

**Published:** 2023-02-07

**Authors:** Mark A. Newpower, Bing‐Hao Chiang, Salahuddin Ahmad, Yong Chen

**Affiliations:** ^1^ Department of Radiation Oncology University of Oklahoma Health Sciences Center Oklahoma City Oklahoma USA; ^2^ Department of Radiation Oncology University of Washington Seattle Washington USA

**Keywords:** machine learning, proton therapy, robustness

## Abstract

The purpose of this work is to assess the robustness of treatment plans when spot delivery errors were predicted with a machine learning (ML) model for intensity modulated proton therapy (IMPT). Over 6000 machine log files from delivered IMPT treatment plans were included in this study. From these log files, over 4.1 ×10^6^ delivered proton spots were used to train the ML model. The presented model was tested and used to predict the spot position as well as the monitor units (MU) per spot, based on the original planning parameters. Two patient plans (one accelerated partial breast irradiation [APBI] and one ependymoma) were recalculated with the predicted spot position/MUs by the ML model and then were re‐analyzed for robustness. Plans with ML predicted spots were less robust than the original clinical plans. In the APBI plan, dosimetric changes to the left lung and heart were not clinically relevant. In the ependymoma plan, the hot spot in the brainstem decreased and the hot spot in the cervical cord increased. Despite these differences, after robustness analysis, both ML spot delivery error plans resulted in >95% of the CTV receiving >95% of the prescription dose. The presented workflow has the potential benefit of including realistic spots information for plan quality checks in IMPT. This work demonstrates that in the two example plans, the plans were still robust when accounting for spot delivery errors as predicted by the ML model.

## 1 INTRODUCTION

In intensity modulated proton therapy (IMPT), a spot pattern is developed in the treatment planning system (TPS) and the intensity of each spot is modulated to achieve a conformal dose to the target while simultaneously sparing critical structures nearby. The proton beam is steered with two orthogonal magnets to control the x and y directions, while the spot's depth is determined by the beam energy. In this technique, the proton system must monitor the position and intensity of each spot during delivery and terminate the beam if the spot position error or monitor units (MUs) error exceed tolerances. Typically, proton treatment delivery systems record and save all relevant data that is measured by the beam monitoring system during patient treatments in the log files. These log files include spot positions, MUs delivered, beam on time and other relevant machine configuration information for every treatment beam.

Log files have been used in different clinical applications in radiation oncology. For example, log files have been used to measure machine performance.^1^, ^2^, ^3^ There is also interest in using log files to complement or even replace patient‐specific quality assurance (QA), a traditionally time and labor‐intensive task.^4^, ^5^ Log files also have been used in volumetric modulated arc therapy (VMAT) patient‐specific QA.^6^, ^7^

Recently there has been significant interest in applying machine learning/artificial intelligence (ML/AI) to radiotherapy.^8^, ^9^ Some of these include dose calculation,^10^, ^11^ automatic contouring of structures,^12^ automated planning,^13^ and prediction of output factors for proton uniform scanning.^14^ The proton system's log files offer a wealth of information about machine performance. The nature of the data in log files readily lends itself to ML applications.

Robust optimization (RO) is a planning strategy used in proton therapy to ensure target coverage is robust in the face of setup and range uncertainties.^15^, ^16^, ^17^ RO typically considers only patient setup and beam range uncertainties. Machine performance is not considered. We propose to determine the robustness of treatment plans when machine delivery errors are included during the plan evaluation process.

The purpose of this work is twofold: (1) to train the ML model to predict spot positions and delivered MUs, based on data from machine log files and (2) to determine what impact these spot delivery errors have on treatment plan robustness. By predicting spot delivery errors with the ML model and analyzing the resulting plan for robustness, we can have a more realistic understanding of the treatment plans delivered to a patient. After robustness analysis of the spot delivery error plans, if CTV coverage is similar to that of the original plan without delivery errors, then these spot delivery errors have a minor effect on plan robustness. However, if there are large differences when spot delivery errors are included, then this may point to an interplay effect between spot delivery errors, patient setup, and stopping power ratio uncertainties.

## 2 MATERIALS AND METHODS

Our institution commissioned the Mevion S250i Hyperscan proton therapy system in January 2019. It is a compact single room pencil beam scanning (PBS) system and is capable of IMPT.^18^ In the Hyperscan system, an adaptive aperture (AA), consisting of multiple pairs of nickel plates, is used to collimate the beam penumbra to enhance OAR sparing. Using the AA, plans can have a static aperture, where the same aperture shape is used for all energy layers, or a dynamic aperture, where the aperture shape is optimized for every energy layer. Our department uses Raystation (Raysearch Laboratories, Stockholm, Sweden) for IMPT treatment planning. Raystation has a powerful scripting interface that allows the user to manipulate most treatment planning data and planning functionalities using the Python programming language. We developed scripts to read and import spot data from log files, enabling us to calculate dose in Raystation from these log files.

Between October 2019 to April 2020, 108 patients treated on the machine had their log files included in this analysis. This work continues previous work in our department of a graphical user interface (GUI) – based Matlab code that analyzes spot positions and MU delivery for a single log file.^19^ This tool has been adapted to analyze multiple log files in an automated fashion.

### 2.1 Correlation between measured and recorded spot positions

A key question in this project is whether the spot positions as recorded by the machine are accurate. As part of weekly QA testing, an evenly spaced 11 × 11 spot position pattern at three gantry angles (0, 90, and 180°) is measured with an IBA Lynx scintillation detector with a spatial resolution of 0.5 mm. The log files from three separate days and gantry angles were collected and compared to the QA data to determine the correlation between the spot positions as measured by the Lynx and as recorded by the proton system and written to log files. The *x* and *y* (crossplane and inplane, respectively) positions of each spot were compared by the average spot position disagreement Δx=x(logfileposition)−x(Lynxmeasuredposition). The root mean square disagreement was calculated by

$$RM\;S_{x}={\left[\left(\frac{1}{n\mathrm{spots}}\right)\times \left(\Delta x_{1}^{2}+\Delta x_{2}^{2}+\cdots \Delta x_{n}^{2}\right)\;\right]}^{1/2}\;$$

### 2.2 Log file analysis and training the ML model

All machine log files were saved as comma separated value (CSV) files. They contain a header with information such as time, date, total prescribed MUs, and other system configuration information. The proton system divides each spot into pulses and repeatedly delivers pulses until the MU per spot has been completely delivered. The body of the log files are organized into rows for each pulse, with over 300 columns of data per pulse. The primary analysis code was written in Matlab version R2018b (MathWorks, Natick, MA). It opened every log file and extracted the pulse's spot index, pulse target and actual positions at isocenter, pulse target and actual MUs, date, snout extension distance, and nominal beam energy. Spot positions were computed by weighing the constituent pulses according to their MUs. Spot position coordinates were recorded in *x* (crossplane) and *y* (inplane). These data were organized into a matrix for input into the Statistics and Machine Learning Toolbox in Matlab R2018b. A shallow feedforward neural network with one hidden layer and five neurons was trained on 70% of the data. The remaining 15% of the data was used for validation and 15% for testing. The Matlab function “trainlm”, a Levenberg‐Marquardt backpropagation algorithm, was used to train the neural network. The Matlab version used for training the model was run on a Dell PowerEdge T420, Dual 6 core Xeon 2.20 Ghz server.

Matlab R2021a was used to develop scripts to extract target spot position, target MU, gantry angle, and snout extension from the DICOM RT Plan file. The data was then converted into a matrix format for use as input into the ML model. Another series of scripts then wrote a “pseudo log file” (PLF) based on the ML model's output. This PLF was then used as input for the Raystation scripts mentioned below.

A series of scripts were developed for use in Raystation (versions 10A and 11A) to extract pulse data in the log files and to create a treatment plan with those parameters. The Mevion S250i Hyperscan system utilizes the AA system to trim the beam penumbra. AA positions from the original plan were applied to the log‐file based plans using another script. Both of the presented patient cases for analysis were treated after the September 2019–April 2020 time window so the spots from those plans were not included in the dataset used to train the ML model. This project received approval from our institutional review board (IRB).

### 2.3 Patient examples

We present two example patient plans in this work, an accelerated partial breast irradiation (APBI) plan and an ependymoma primary plan. The APBI patient received 3000 cGy(RBE) in five fractions from two anterior beams. The ependymoma patient received 5040 cGy(RBE) in 28 fractions, with six beams (RAO, LAO, LPO, RPO, LL, and RL) in the primary plan, and then a boost of 900 cGy(RBE) in five fractions with the same beam geometry. In both cases, the dose was normalized such that 99% of the CTV received the prescription dose. The APBI plan utilized a static aperture and the ependymoma plan had a dynamic aperture. Both plans utilized RO to ensure coverage for the CTV. The APBI robustness optimization settings were an isotropic uncertainty of ± 5 mm and a density uncertainty of ± 3.5%. The ependymoma plan's RO settings were an isotropic uncertainty of ± 3 mm and a density uncertainty of ± 3.5%. Four plans were used in this study: (1) the clinical plan for the APBI patient, (2) the clinical plan for the ependymoma patient, (3) the ML predicted spot delivery plan on the APBI patient, and (4) the ML predicted spot delivery plan on the ependymoma patient. Robustness analysis was performed on each plan by computing 16 scenarios for each plan: ± isotropic shifts in right‐left, inferior‐superior and posterior‐anterior, and ± 3.5% density shifts. The plans were shifted ± 5 mm in for the APBI plan and ± 3 mm in the ependymoma plan. In this study, we consider plans to be robust if at least 95% of the CTV receives at least 95% of the prescription dose.

## 3 RESULTS

### 3.1 Correlation between measured and recorded spot positions

Table 1 lists the summary of the agreement between the measured spot positions and log file spot positions. The spot position error as a function of gantry angle was fully investigated in our clinic during commissioning. Overall, the result indicated good agreement between the measured spot positions and recorded spot positions from the log file. For the 11 ×  11 spot pattern, the mean Δ between the measured and recorded spot positions was less than 0.5 mm in the *x* (crossplane) direction and was close to 1.0 mm in the *y* (inplane) direction. The distribution of Δ for each spot position followed a Gaussian distribution.

**TABLE 1:** Correlation between spot positions as measured on the Lynx and recorded by the system and written to the log files at three gantry angles

	**Gantry angle**		
**Parameter**	**0°**	**90°**	**180°**
$\bar{\Delta x}$	0.119 ± 0.251	−0.366 ± 0.451	0.357 ± 0.237
$\bar{\Delta y}$	−1.281 ± 0.269	−1.081 ± 0.315	−0.650 ± 0.194
RMS_x_	0.277	0.579	0.428
RMS_y_	1.309	1.126	0.678
RMS_xy_	1.338	1.266	0.802

*Note*: All distances are in mm.

### 3.2 Log file analysis and training the ML model

**FIGURE 1:**
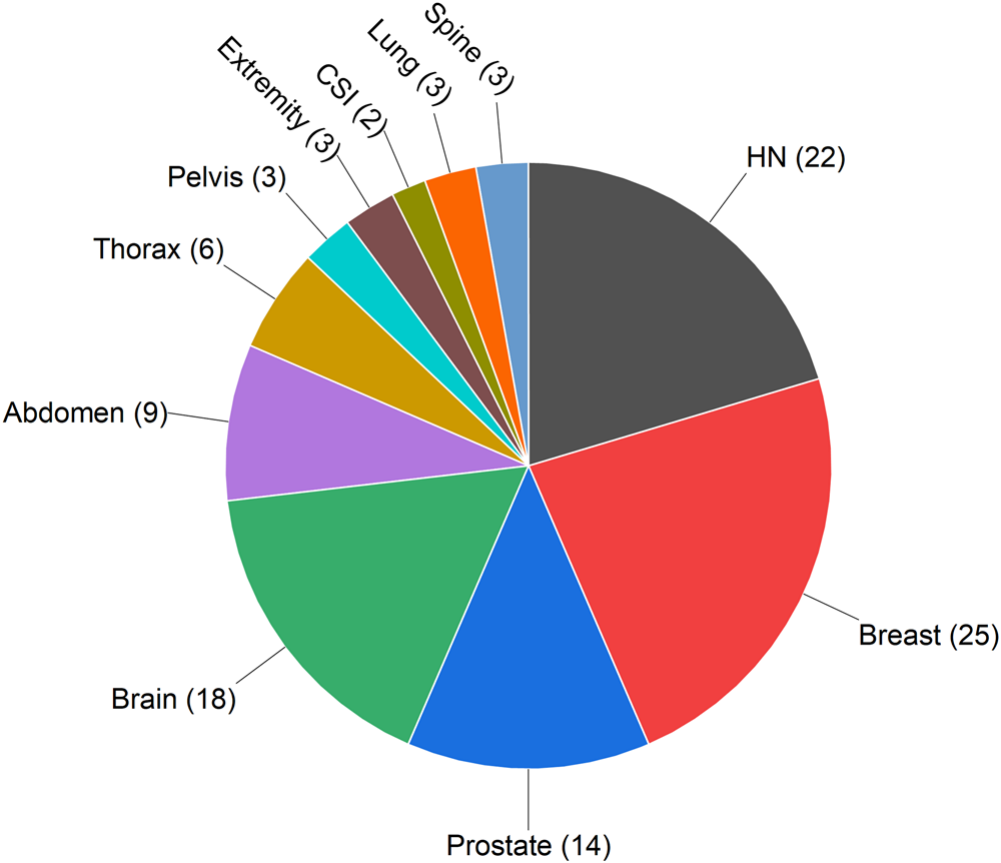
A pie chart breaking down the patient data included in this study by disease site. The number of patients for that disease site is shown in parentheses. CSI, craniospinal irradiation; HN, head and neck.

Figure 1 shows the breakdown of patients by disease site. In total over 6000 log files were mined to yield delivery data on 4.1 ×  10^6^ spots. These spots were used as input to train the ML model. The mean‐squared error of the ML model fit on the testing data was 0.301 and the *R*2 value was 0.9997. Spot position delivery errors, or the difference between the target spot position and the actual delivered position recorded in the log file, followed a Gaussian distribution.

### 3.3 Patient examples

In both cases, the ML model predicted some spot positions outside the AA. These spots outside the AA were deleted from the plan considering Raystation did not allow the user to calculate dose with spots whose centroid were outside the AA. As a result, the CTV was underdosed on the order of 1 cGy(RBE) in both ML spot prediction plans. One spot was deleted from the APBI plan and one from the ependymoma plan.

**FIGURE 2:**
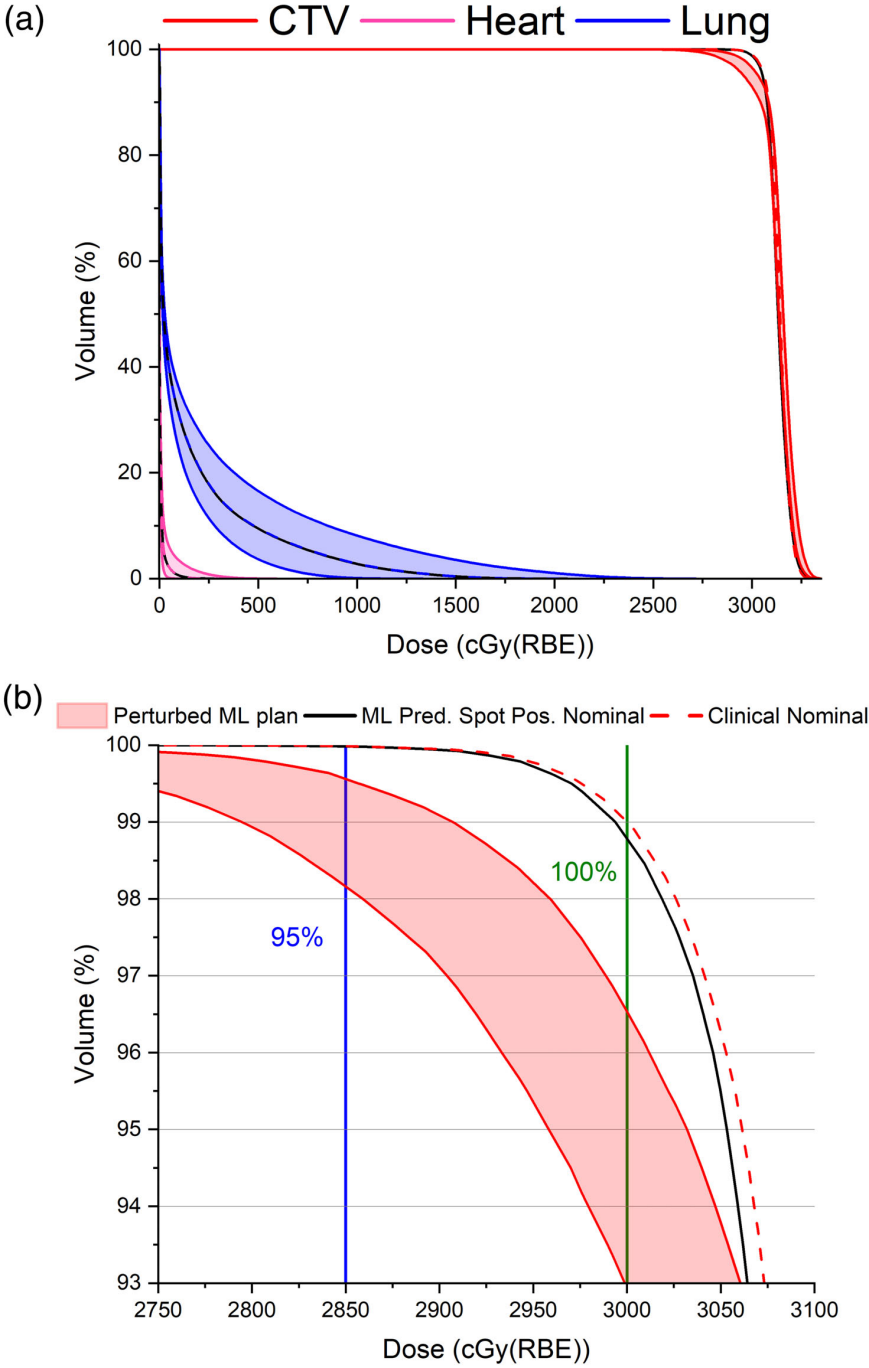
(a) Banded DVH curves for the ML spot predicted APBI plan. The dashed colored lines are the clinical nominal cases for those ROIs. The solid black lines are the ML spot predicted nominal plans for those ROIs. (b) Close up from (a). The blue vertical line represents 95% of the prescription dose. The green vertical line represents 100% of the prescription dose. All plans, even perturbed ML spot delivery plans, resulted in >95% of the CTV receiving >95% of the prescription dose

**FIGURE 3:**
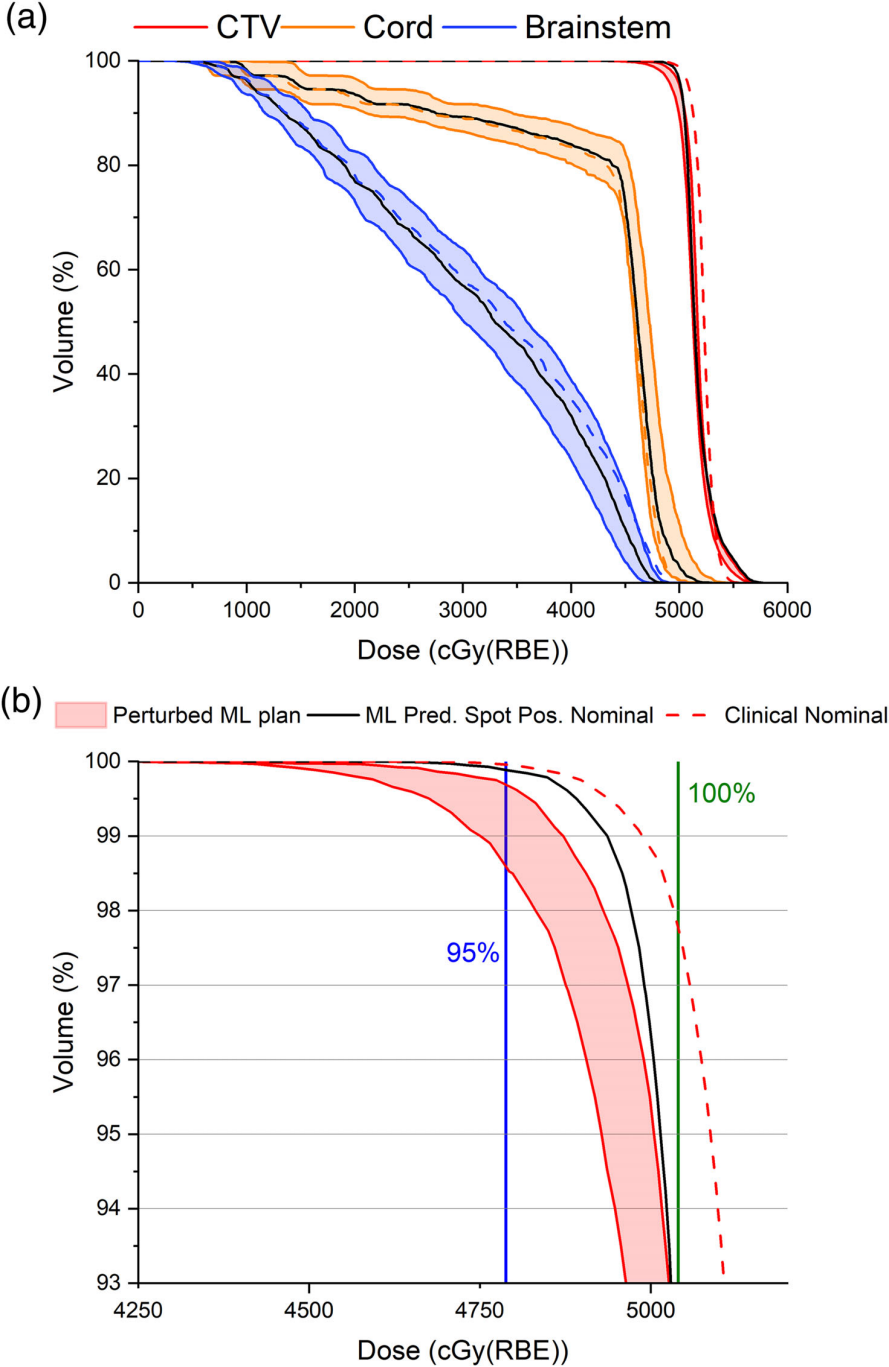
DVH curves for the ML spot predicted ependymoma plan. The dashed colored lines are the clinical nominal cases for those ROIs. The solid black lines are the ML spot predicted nominal plans for those ROIs. (b) Close up from (a). The blue vertical line represents 95% of the prescription dose. The green vertical line represents 100% of the prescription dose. All plans, even perturbed ML spot delivery plans, resulted in >95% of the CTV receiving >95% of the prescription dose

As shown on the DVH in Figures 2 and 3, four curves include: (1) the nominal clinical plan (no perturbation), (2) the nominal ML plan (no perturbation), (3) the minimum DVH, and (4) maximum DVH curves on the perturbed ML plan were drawn to facilitate comparison between the ML‐predicted spot position plan and the original plan. The maximum and minimum DVH curves form the boundaries of the banded DVH for each structure. The nominal clinical and nominal ML curves tended to fall within the band for each structure. In Tables 2 and 3, the DVH indices were generated by reviewing every scenario in the robustness evaluation. For instance, in the APBI case, the coldest DVH curve for the heart was not necessarily the coldest DVH curve for the spinal cord. Analyzing the data in this way gives the reader a better sense of all possible scenarios under these robustness evaluation criteria. Select DVH indices are given in Tables 2 (CTV coverage) and 3 (OAR doses).

**TABLE 2:** Summary of robustness analysis of CTV coverage for the APBI and ependymoma plans

**CTV coverage**					
		**Clinical plan**		**ML predicted spots**	
	**Scenario**	**D99**	**D0.03cc**	**D99**	**D0.03cc**
APBI	CTV min	2846 (94.9%)	3258 (108.6%)	2796 (93.2%)	3262 (108.7%)
	Clinical nominal	2991 (99.7%)	3284 (109.5%)	–	–
	ML nominal	–	–	2988 (99.6%)	3306 (110.2%)
	CTV max	2895 (96.5%)	3342 (111.4%)	2924 (97.5%)	3345 (111.5%)
Ependymoma	CTV min	4802 (95.3%)	5477 (108.7%)	4746 (94.2%)	5673 (112.6%)
	Clinical nominal	4988 (98.97%)	5330 (105.8%)	–	–
	ML nominal	–	–	4936 (97.9%)	5751 (114.1%)
	CTV max	4931 (97.8%)	5577 (110.7%)	4872 (94.8%)	5805 (115.2%)

*Note*: All values are in cGy(RBE). Values in parentheses are percentages of the prescription dose, which was 3000 cGy(RBE) for the APBI CTV and 5040 cGy(RBE) for the ependymoma CTV.

**TABLE 3:** Summary of robustness analysis of OAR doses for the APBI and ependymoma plan

**OAR doses**			
	**Scenario**	**Clinical plan**	**ML predicted spots**
		Dose to 950 cc (lung)	Dose to 950 cc (lung)
APBI	Lung min	15	15 (+0)
	Clinical nominal	20	–
	ML nominal	–	24
	Lung max	38	38 (+0)
		Dose to 5 cc (Brainstem)	Dose to 5 cc (Brainstem)
Ependymoma	Brainstem min	4132	4052 (−80)
	Clinical nominal	4360	–
	ML nominal	–	4275
	Brainstem max	4560	4469 (−91)
		Dose to 5 cc (Cord)	Dose to 5 cc (Cord)
Ependymoma	Cord min	2407	2537 (+130)
	Clinical nominal	3055	–
	ML nominal	–	3160
	Cord max	3684	3806 (+122)

*Note*: All values are in cGy(RBE). The values in parentheses in the ML predicted spots column is the difference in that DVH between the clinical plan and the ML predicted spot plan.

#### 3.3.1 APBI

Robustness evaluation between the clinical plan and the ML spot position plans showed very similar DVHs. Comparisons between clinical plan and the ML spot position plan are noted in Tables 2 and 3. Three volumes are included in the dose volume histogram shown in Figure 2: CTV, left lung and heart. In the nominal ML scenario, 99% of the CTV (D99%) received 99.6% of the clinical dose, or a slight underdose. The worst‐case scenario for CTV coverage in the ML predicted spot plan was 93.2% of the prescription dose. The left lung had the largest variation between minimum and maximum DVH curves, but each perturbed scenario was well within dose constraints. The banded DVH for the heart showed small variations but each scenario was well within the Timmerman dose constraints.^20^ Robustness evaluation on the CTV between the clinical plan and the ML spot position plans showed very similar DVHs, with differences usually <1% of the prescription dose as shown in Table 2. In Table 3, the dose to 950 cc of lung is shown for the various scenarios. When comparing the ML nominal and clinical nominal plans, the differences across each scenario were minor for the lung in the APBI plan.

#### 3.3.2 Ependymoma

Three volumes are included in the DVH shown in Figure 3: CTV, cervical spinal cord, and brainstem. In the nominal clinical scenario, D99% was 98.97% of the prescription dose. In the nominal ML scenario, D99% was 97.9% of the clinical dose. Robustness evaluation on the CTV between the clinical plan and the ML spot position plans showed very similar DVHs, with differences usually <1% of the prescription dose as shown in Table 2. The worst‐case scenario for CTV coverage in the ML predicted spot plan was 94.2% of the prescription dose.

The dose differences to the cervical cord and brainstem are summarized in Table 3. In the ML predicted spot plan, the minimum DVH curve to 5 cc of the brainstem robustness analysis decreased by 80 cGy(RBE) over the clinical plan. A decrease of 91 cGy(RBE) also observed on the brainstem maximum DVH. The ML predicted spot plan cervical cord minimum DVH curve increased by 130 cGy(RBE) over the clinical plan and the cord maximum DVH curve also increased, by 122 cGy(RBE).

## 4 DISCUSSION

### 4.1 Correlation between measured and recorded spot positions

Overall, the agreement between the spot position as recorded by the Lynx scintillation device and the machine log files was on the order of ± 1 mm in the y (inplane) direction and less than ± 1 mm in the *x* (crossplane) direction according to Table 1. The position discrepancy was systematically greater in the y‐direction than the x‐direction. For all spot positions and gantry angles, the Δ between the log file and Lynx position followed a Gaussian distribution. A drawback of this study was the inability of the Lynx to differentiate between random and systematic spot delivery errors. However, all spot position errors were within 1 mm indicated agreement between measured and recorded spot positions. Based on these results we are confident the spot positions reported in the log files were accurate.

### 4.2 Log file analysis and training the ML model

4.1×10^6^ spots were used to train the ML model. These spots included a large range of gantry angles, snout extensions, and beam energies. This large dataset was appropriate to train a generalized ML model. The model's coefficient of correlation was very close to unity, indicating the model was able to predict the spot position and MU well.

### 4.3 Patient examples

In both plans, the D99% for the CTV in the clinical nominal scenario was slightly less than 100%. For the APBI case, the clinical nominal D99% was 2991 cGy(RBE), and not 3000 cGy(RBE), as was the prescription. A similar, slight reduction in D99% was seen for the clinical nominal ependymoma case. These differences can be attributed to a different version of the Monte Carlo dose calculation engine used between the Raystation versions used to create the original plan (v10A) and the current version used to complete this analysis (v11A).

In both example plans, the ML model predicted spots, which were outside the AA, and were removed. Raystation does not allow the user to calculate dose with spots whose centroid are outside the edge of the field defined by the AA, so these spots were deleted from the plan. One spot was deleted from the APBI plan and one from the ependymoma plan. However, the dose contribution from a single spot is small. We investigated the impact of those spot deletions by making a copy of the clinical plan, deleting the spot that was predicted to be blocked by the AA, and recalculated the dose. We found D99% of CTV decreased by 1 cGy(RBE) in each case; it decreased from 2991 to 2990 cGy(RBE) in the APBI plan, and decreased from 4988 to 4987 cGy(RBE) in the ependymoma plan. From this, we conclude that the decreased CTV coverage in the ML predicted plans are mostly due to spot delivery errors, not the deletion of a spot. Although the spots were just beyond the border of the AA, usually within ∼1 mm of the edge, the S250i Hyperscan system's spot sizes are relatively large, on the order of σ≈1 cm at 150 MeV at isocenter in air. Therefore, even a spot whose centroid is ∼1 mm beyond the edge of the AA would still contribute some dose to the patient. Thus, the patient is most likely getting more dose than is predicted by the TPS for these spots. For both plans, the minimum CTV coverage scenario was slightly worse with the ML predicted plans. This can partly be attributed to the small number of spots that were unable to be calculated due to their position behind the AA, and mostly due to spot delivery errors predicted by the ML model.

Both the APBI and ependymoma ML spot predicted plans were analyzed for robustness. All scenarios in both plans resulted in at least 95% of the prescription dose covering at least 95% of the CTV. From this, we conclude that both plans are robust, even when taking spot delivery errors into account. In the APBI plan, CTV D99% decreased from 2991 cGy(RBE) to 2988 cGy(RBE) when considering the ML predicted spots versus the nominal clinical plan (Table 2). This is due to slight spot delivery errors, and those errors, cumulatively, lead to slightly diminished CTV coverage. This effect was much larger, a D99% decrease of 52 cGy(RBE) in the ependymoma plan. Both plans were optimized using multi‐field optimization (MFO). However, the resulting dose distribution for the APBI plan was still very similar to what a single field optimization (SFO) plan would yield: dose split almost evenly between the two fields. These plans produce dose distributions per field that are relatively homogeneous and therefore insensitive to spot delivery errors. In contrast, the ependymoma plan used 6 beam angles. The resulting dose distribution per field was highly heterogenous, a common attribute of MFO plans. Spot perturbations in these plans are more likely to create hot and cold spots since each beam's dose distribution is highly heterogeneous.

Regarding OAR doses, in the APBI case, there were some variations in heart and lung doses, but these were well outside of constraints. In the ependymoma plan, there were scenarios when significant hot or cold spots appeared. These occurred in the spinal cord (hot spot) and brainstem (cold spot). These hot/cold spots could be clinically relevant if those OARs are receiving doses near constraints. Further analysis could identify which spots are most prone to delivery errors that lead to hot/cold spots, and these spots could be modified by the treatment planner if necessary.

This type of analysis, predicting the dosimetric impact of spot delivery errors in IMPT plans using the ML model, and those errors' effect on plan robustness, has not been published to our knowledge. Typically, robustness analysis does not consider machine delivery errors. This work shows that even when including these errors, plan robustness is minimally affected. In future studies, it would be useful to study more treatment plans and use this ML model to identify more instances where machine delivery errors lead to either significant reductions in robustness, or the creation of hot or cold spots in OARs.

This study did not consider daily variations in spot delivery, only consistent delivery errors as predicted by the ML model. Daily spot delivery errors will likely be similar to but not exactly matching the predictions of the ML model. Based on our in‐depth analysis of spot delivery errors, we expect daily spot position delivery errors would follow a Gaussian distribution, whose mean is on the order of 1 mm or less from the planned spot position. An interplay effect between spot delivery errors and setup and stopping power ratio uncertainty was observed to be minimal in this study. After robustness analysis was complete, all plans met a minimum robustness standard of at least 95% of the CTV receiving at least 95 % of the prescription dose.

This study presents a novel method to utilize ML models in clinical practice. A clinic could follow this same methodology by gathering their machine log files and using them to create the ML model to predict spot delivery errors, then assess the impact of these errors on their own treatment plans. An open question is, to what degree are these treatment delivery errors impacting treatment plan quality across different proton therapy systems? TPS vendors could also implement the presented workflow into their TPSs as part of robustness analysis. If machine delivery errors were found to have clinically relevant impacts, then clinicians and physicists would be interested in evaluating these impacts on treatment plans in their clinics.

## 5 CONCLUSION

In this work, we present the ML model, trained from the Mevion S250i Hyperscan's treatment log files, which can predict the delivered spot position and MU in an IMPT plan. Two plans were used as examples, an APBI plan and an ependymoma primary plan. For these plans, their planned spot positions and MUs were used as input for the ML model. The output of the ML model, the predicted spot positions and MUs were then imported into Raystation (v11A), and the dose calculated. Both plans were then analyzed for robustness.

Based on the results of this analysis we conclude that despite spot delivery errors predicted by the ML model, both the APBI and ependymoma plans were robust. This work demonstrates the utility of the ML model that can be used to gain a more comprehensive understanding of the IMPT plans delivered to patients.

## AUTHOR CONTRIBUTIONS

The authors confirm their contribution to the paper as follows. Study conception and design: Mark Newpower and Yong Chen. Acquisition, collection, and assembly of data: Mark Newpower, Bing‐Hao Chiang, and Yong Chen. Analysis and interpretation of results: Mark Newpower, Yong Chen, and Salahuddin Ahmad. Draft manuscript preparation: Mark Newpower, Bing‐Hao Chiang, Salahuddin Ahmad, and Yong Chen. Final approval of the version to be published: Mark Newpower, Bing‐Hao Chiang, Yong Chen, and Salahuddin Ahmad. All authors reviewed the results and approved the final version of the manuscript.

## CONFLICT OF INTEREST

All authors have read and agreed to the published version of the manuscript, and have no conflicts of interest to declare.
